# Characterization of Dominant Heterotrophic Flagellates in Anaerobic Digesters Using Combined Culture-based and Metabarcoding Approaches

**DOI:** 10.1007/s00248-026-02768-5

**Published:** 2026-04-15

**Authors:** Hyeon Been Lee, Aaron A. Heiss, Dong Hyuk Jeong, Jinho Cho, ChangWeon Lee, Byung Cheol Cho, Jong Soo Park

**Affiliations:** 1 Department of Oceanography, Kyungpook National University, Daegu, Republic of Korea; 2 Microbial Oceanography Laboratory, Kyungpook Institute of Oceanography, Kyungpook National University, Daegu, Republic of Korea; 3 SK Incheon Petrochem Co., Ltd, Incheon, Republic of Korea; 4 School of Earth and Environmental Sciences, Seoul National University, Seoul, Republic of Korea

**Keywords:** Anaerobic digester, Heterotrophic flagellates, *Tetratrichomonas*, Metabarcoding

## Abstract

While bacteria and archaea are regarded as major components of anaerobic ecosystems, eukaryotes have received less attention, especially in engineered ecosystems such as waste reclamation plants. Previous work on these environments has tended either to focus on individual organism strains in culture or on metabarcoding sequence analysis of uncultured material; we employed both approaches. Interestingly, the culture-independent analysis of a full-scale anaerobic digester mostly detected aerobic eukaryotes that originated directly from feed material, such as cabbage, sweet potato, aerobic fungi, and mites. Remarkably, the anaerobic protist *Tetratrichomonas* was the dominant organism in all samples, consistently present but showing a more pronounced peak in April than in other months; its bloom is likely associated with increased levels of volatile fatty acids and biogas production. Also, we surveyed the protist communities in full-scale anaerobic digesters microscopically, from which we isolated novel heterotrophic flagellates; based on sequence identity, molecular phylogeny, and morphology, we identify as these *Tetratrichomonas* spp. Although neither culture-dependent nor culture-independent approaches give a complete picture of the communities within these ecosystems, both suggest that *Tetratrichomonas* may be a key genus in Korean mesophilic (36.6–38.9 ˚C) anaerobic digesters treating a mixture of livestock manure, food waste, and sewage sludge.

**Supplementary Information:**

The online version contains supplementary material available at 10.1007/s00248-026-02768-5.

## Introduction

While all known eukaryotes have an aerobic ancestry, some have developed a variety of adaptations to live in anaerobic environments [1]. There is likewise a substantial amount of variation in anaerobic habitats, which include such engineered ecosystems as the treatment plants for agricultural and municipal waste. Anaerobic digestion is the biological degradation of organic matter under anaerobic conditions, converting diverse substrates such as animal manure, wastewater sludge, and food waste into biogas. It represents a highly flexible biotechnology, such that anaerobic digesters (or reactors) can be operated in various configurations and modes depending on substrate characteristics and technological setup. In continuously operated systems, process conditions can remain quite steady in terms of (e.g.) temperature, pH, and salinity; the primary variable affecting the biota in them is the feed material that they process, and that too can be controlled. However, variations in operational factors do occur, and in particular, newly set-up digesters take some time to establish mature ecosystems [2]. Nevertheless, the number of variables that must be accounted for in determining the cause and effect of changes in digester community composition is much smaller than in most other ecosystems, especially naturally occurring ones. This simplicity facilitates the maintenance of stable system performance.

The eukaryotes occupying these anaerobic environments have received little scientific attention [3, 4]. While prokaryotes are responsible for key reactions in anaerobic digesters, the role of eukaryotes should not be disregarded [5]. Although a variety of eukaryotes have been identified in anaerobic digesters [4, 6–9], seasonal distribution patterns and comparisons of diversity between eukaryotes capable of remaining viable under anaerobic conditions (on the one hand) and aerobic eukaryotes unable to survive in such environments (on the other) remain poorly studied.

Traditionally, eukaryotes from all environments have been identified morphologically. This approach relies heavily on individual expertise and experience for accurate results, and generally focuses on a small number of eukaryotes. Nevertheless, culture-based approaches can be invaluable not only for identification of organisms but also for investigating their metabolic capacities. To our knowledge, Hirakata et al. [6] was the only previous culture-based study of a full-scale anaerobic digester. Consequently, the diversity and roles of viable eukaryotes in anaerobic digesters remain poorly understood from a culture-dependent perspective.

Next-generation sequencing (NGS) has allowed for large-scale ‘metabarcoding’ surveys of an environment using probes for specific regions of the genome (e.g., [10]). This yields two types of data: the number of amplicon sequence variants (ASVs), which corresponds to the diversity of sequences and thus the number of taxa sampled, and the number of reads of each ASV, which is a function of the number of corresponding individual organisms sequenced. It must be noted, however, that gene copy numbers vary, such that read counts are not necessarily an accurate proxy for the number of cells sampled (e.g., [11]). Another potential drawback to the use of NGS is a limitation of the length of sequence that can be reliably obtained. The most-used targets for metabarcoding are the ~ 300–500-base-long V4 and V9 hypervariable regions of the small-subunit (SSU or 18S) ribosomal RNA gene (e.g., [4, 12, 13]). Each region has its advantages and drawbacks, however: in particular, the V4 region is generally more specific, while the V9 region samples wider diversity [12, 13].

Our study represents a pilot investigation of microbial eukaryotes in Korean mesophilic anaerobic digesters, in which we hypothesized that: (1) as reported in previous studies in other regions [4, 6–9], microbial eukaryotes are present in Korean anaerobic digesters; (2) some eukaryotes are inactive or dead cells introduced through feed material, whereas others are metabolically active and capable of growing within the digester environment; (3) active eukaryotes constitute a substantial component of the overall eukaryotic community; and (4) their presence is correlated with digester performance parameters, such as volatile fatty acid (VFA) and biogas production. To test these hypotheses, we combined culture-based and metabarcoding approaches to assess the eukaryotic diversity within continuously stirred tank reactors (CSTRs), one of the more common types of anaerobic digesters [14]. We assessed the eukaryotic microbial community in the CSTR located in Geumsan, Republic of Korea, using NGS metabarcoding of the V9 hypervariable region, based on three sampling events conducted over ten months. Intriguingly, the anaerobic heterotrophic flagellate *Tetratrichomonas* was consistently detected in all metabarcoding datasets. To further investigate active eukaryotes in these systems, we examined the same material microscopically and successfully established a monoprotistan culture of *Tetratrichomonas* from the Geumsan digester. Subsequently, we established a similar monoprotistan culture from another CSTR located in Icheon, Republic of Korea, thereby expanding the distribution range of this genus in anaerobic digesters. The recurrent detection of *Tetratrichomonas* suggests that it may serve as a crucial potential component of Korean mesophilic anaerobic digesters. Therefore, the systematic monitoring of *Tetratrichomonas* in anaerobic digesters worldwide is recommended in the near future to clarify its functional role and its implications for digester performance and management.

## Methods

### Operational Factors

Operational factors were monitored between January 2023 and May 2024 in the CSTR located in Geumsan, Republic of Korea (“GS”; 36°5’54” N, 127°30’50” E; start-up date: November 8, 2022; reactor volume: 90 m^3^; residence time: 40 days; mesophilic operation from 36.6℃ to 38.9℃). Measurements of temperature, pH, VFAs, alkalinity, total nitrogen (TN), total solids (TS), volatile solids (VS), and biogas were recorded using in-situ sensors and integrated monitoring systems. The feed material of the anaerobic digester in Geumsan was co-digested, consisting of a mixture of livestock manure, food waste, and sewage sludge at an approximate ratio of 6:1:1.

### Metabarcoding

**Fig. 1:**
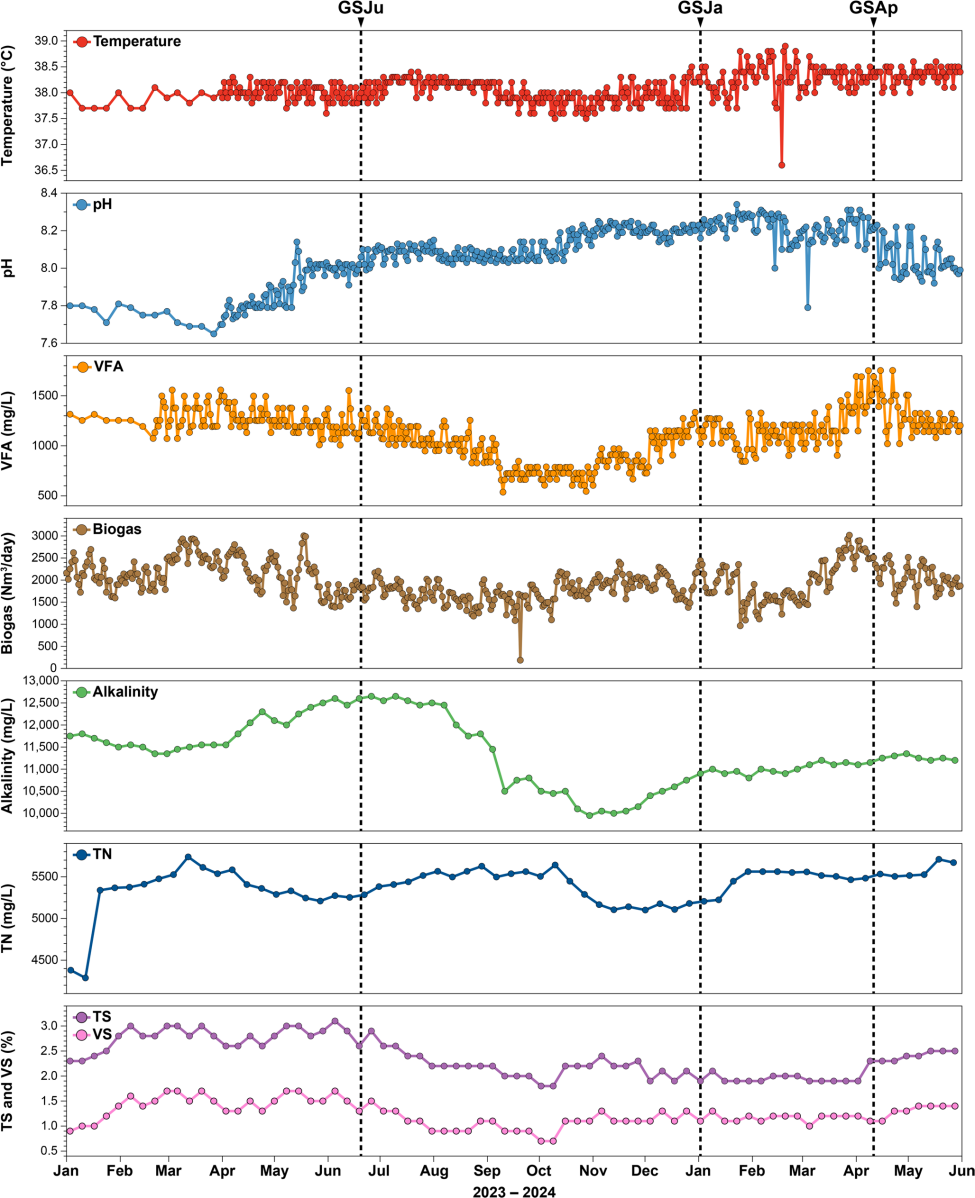
Operational factors of anaerobic digesters in Geumsan between January 2023 and May 2024. Temperature, pH, volatile fatty acid (VFA), and biogas measurements were taken on a daily basis; alkalinity, total nitrogen (TN), total solids (TS), and volatile solids (VS) were sampled weekly. Sampling points for metabarcoding are indicated by dashed vertical lines

To analyze the in-situ biodiversity in an anaerobic digester, 500 mL of anaerobic sludge samples were collected from the reactor located in Geumsan on three occasions: June 20, 2023 (“GSJu”), and January 2 (“GSJa”) and April 11 (“GSAp”), 2024. These three sampling points were selected to capture potential seasonal variation in feed material composition, as June, January, and April correspond to summer, winter, and spring in Korea, respectively (see Discussion section). GSJu, GSJa, and GSAp had salinities of 13, 10, and 13 PSU, respectively. Aside from salinity, all chemical assays, including those for the sampled sludge as well as spanning the entire operating period of the anaerobic digester, were provided by the treatment facility (Fig. 1).

Samples were centrifuged at 1,000 × *g* for 10 min. The supernatant was filtered through 0.65-µm-pore-size PVDF (Durapore: Merck Millipore, Darmstadt, Germany) membrane filters using a vacuum pump. These filters were then cut into small pieces, from which nucleic acids were extracted using the DNeasy Blood and Tissue Kit (Qiagen, Hilden, Germany) according to the manufacturer’s instructions. Nucleic acid was also extracted from the pellet using the DNeasy PowerMax Soil Kit (Qiagen). The DNA samples obtained from the supernatant and pellet were then combined and purified using the QIAquick Nucleotide Removal Kit (Qiagen). The extracted DNA concentrations were measured with a Qubit 4 Fluorometer and Qubit 1× dsDNA High Sensitivity (HS) Assay Kit (Invitrogen, Thermo Fisher Scientific, Waltham, MA, USA), yielding DNA concentrations of 4,970, 3,450, and 708 ng/µL for GSJu, GSJa, and GSAp, respectively.

The V9 hypervariable region of the 18S ribosomal RNA gene, which is known to provide broad taxonomic coverage across diverse eukaryotic lineages [12], was amplified via PCR using a standard primer set (V9 forward: 5’-CCCTGCCHTTTGTACACAC-3’; V9 reverse: 5’-CCTTCYGCAGGTTCACCTAC-3’; [12, 15]) augmented with P5 and P7 adapter sequences (Illumina Inc., San Diego, CA, USA). Each sample was amplified in triplicate. Amplification occurred in 23.5-µl volumes using Herculase II Fusion DNA polymerase (Agilent, Waldbronn, Germany), with 1.25 µL of each 10 µM primer and 2 µL of extracted DNA, and was performed on a Biometra TRIO thermal cycler (Analytik Jena, Jena, Germany). The PCR program included 30 cycles, with an annealing temperature of 57 °C and an extension time of 90 s, and with an initial denaturing step at 94 °C for 3 min and a final hold at 72 °C for 10 min.

Further processing and analysis were performed by Macrogen, Inc. (Seoul, Republic of Korea). Initial PCR products were purified and concentrated using Agencourt AMPure XP beads (Beckman Coulter, Brea, CA, USA). A second PCR, which attached dual index primers and sequencing adapters, was performed using the Nextera XT Index Kit (Illumina Inc., San Diego, CA, USA). Amplicon libraries were further purified using Agencourt AMPure XP beads and sequenced using the Illumina MiSeq platform (Illumina). The raw sequencing data were deposited at NCBI as BioProject PRJNA1247748.

After sequencing, Cutadapt v3.2 [16] was used first to remove adapter and primer sequences from the raw data, and then to trim forward and reverse reads to lengths of 250 and 200 bp, respectively. To generate amplicon sequence variants (ASVs), DADA2 v1.18.0 [17] was used to exclude reads with expected errors of 2 or more bases, to denoise erroneous reads based on an established error model, to merge paired-end reads by overlapping, and to remove chimeric sequences (the latter specifically using the ‘removeBimeraDenovo’ function). Each ASV was assigned to the organism with the highest similarity in the GenBank ‘nt’ reference database using BLAST+ v2.9.0 [18], with criteria for acceptance of query including both > 85% coverage and > 85% identity.

To assess sampling sufficiency and compare species richness between samples, rarefaction analysis was performed with DataGraph 4.7 (Fig. S1).

### Isolation and Culturing

To isolate heterotrophic protists, raw samples of 50 mL of anaerobic sludge were collected from the reactor in Geumsan (“GS”, sampled on April 4, June 20, and August 29, 2023), and in addition from another CSTR in Icheon, Republic of Korea (“IC”; 37°8’21” N, 127°29’6” E; start-up date: December 2021; reactor volume: 99 m^3^; residence time: 30–40 days; mesophilic operation; feed: a co-digested mixture of livestock manure and food waste at approximately a 7:3 ratio; sampled on September 19, 2023). The GS samples collected in April, June, and August had temperatures of 37.9, 38.0, and 38.2 °C and salinities of 13, 13, and 17 practical salinity units (PSU), respectively, whereas the IC sample had a temperature of 36 °C and a salinity of 19 PSU. Both active and encysted eukaryotes were observed in raw samples by phase contrast and differential interference contrast microscopy. Strains SK002_GS, SK003_GS, and SK004_GS were isolated from the GS samples collected in April, June, and August, respectively, while strain SK006_IC was isolated from the IC sample. All strains were enriched in 10 PSU T/F medium (0.5 g yeast extract, 1 g tryptone, 1.4 g K_2_HPO_4_, 0.2 g KH_2_PO_4_, 10 g NaCl, and 30 mL heat-inactivated horse serum per liter) containing 1 mM sodium pyruvate (final conc.), as this medium supported the best growth among several tested formulations. Monoprotistan cultures were established by 3–4 cycles of serial dilution at a 1:9 ratio. Cultures were maintained at 37 °C in the same media in tightly capped 15-mL conical tubes with less than 1 mL airspace, and transferred monthly.

### Molecular Phylogenetics

DNA was extracted from the cultures using the DNeasy Blood and Tissue Kit (Qiagen) according to the manufacturer’s instructions. 18S rRNA gene sequences were amplified via PCR using general-eukaryote primers EukA (5’-AACCTGGTTGATCCTGCCAGT-3’) and EukB (5’-TGATCCTTCTGCAGGTTCACCTAC-3’) [19]. Amplification was performed using TaKaRa *Taq* polymerase (TaKaRa Bio, Shiga, Japan) in a Biometra TRIO thermal cycler (Analytik Jena). Cycling conditions included 40 cycles, with an annealing temperature of 55 °C and a 3 min extension time, and an initial denaturing step of 94 °C for 5 min and a final extension step for 20 min at 72 °C. Amplicons were purified and sequenced directly using the Sanger method (Macrogen Inc., Seoul, Republic of Korea). The 18S rRNA gene sequences from strains SK004_GS and SK006_IC were deposited in GenBank under the accession codes PV473671 and PV473672, respectively. Sequence identity was assessed using BLAST [20] against the GenBank ‘nt’ database.

A reference alignment was generated by combining sequences of strains SK004_GS and SK006_IC with sequences representing 45 members of Trichomonadidae and three of Lacusteriidae (sensu [21]), resulting in 50 total taxa; all sequences were aligned using MAFFT v7 [22] with the L-INS-i algorithm. This reference alignment was used to infer a maximum-likelihood (ML) backbone tree in IQ-TREE v1.6.12 [23] under the GTR + F+I+G4 model selected by the best-fit model test option (-m TEST), with branch support assessed from 1,000 ultrafast bootstrap replicates. Bayesian inference was performed using MrBayes 3.2.7a [24] with 2 parallel MCMCMC runs of four chains each (heating parameter = 0.1), sampled every 1,000 generations and discarding the initial 30% as burn-in; convergence, defined as an average standard deviation of < 0.01 between runs, was reached after approximately 7,000,000 generations. Six *Tetratrichomonas*-related ASVs recovered from metabarcoding were subsequently incorporated by aligning them to the reference alignment using MAFFT with the ‘-addfragments’ option [22]. Phylogenetic placement of these ASVs onto the backbone tree was then performed using EPA-ng [25] under the GTR + F+I+G4 model inferred by IQ-TREE.

### Correlation Analysis

To examine the relationships between environmental factors and eukaryotic taxa, pairwise Spearman’s rank correlation coefficients were calculated between eight environmental variables (temperature, pH, VFAs, biogas, alkalinity, TN, TS, and VS) and the 18 eukaryotic genera with more than 1% abundance; other tests were inappropriate due to the limited number of samples. For each sampling point, environmental variables were averaged over the 30 days preceding the sampling date and used as representative values for correlation analysis. Because only three sampling points were available, the assumptions of normality required for parametric correlation analysis could not be satisfied; therefore, the non-parametric Spearman method was applied. Spearman correlation coefficients (ρ) were computed in Python using the ‘scipy.stats.spearmanr’ function [26], and the resulting correlation matrix was visualized as a heatmap using the ‘seaborn’ package [27]. Given the limited number of observations (*n* = 3), *p*-values were not interpreted for statistical significance; instead, the correlation coefficients were used to assess possible monotonic patterns between environmental variables and eukaryotic community composition, rather than to infer strong relationships between them.

## Results

### Operational Factors of Anaerobic Digester in Geumsan

Temperature ranged from 36.6 °C to 38.9 °C, and pH from 7.7 to 8.3, between January 2023 and May 2024 (Fig. 1). Volatile fatty acid (VFA) and biogas production showed similar temporal trends, with pronounced peaks around April in both 2023 and 2024 (Fig. 1). Alkalinity showed a relatively high peak in summer 2023 (Fig. 1). Total nitrogen (TN) was not observed to have a seasonal pattern (Fig. 1). Total solids (TS) and volatile solids (VS) gradually decreased from June 2023, without any apparent seasonal variation (Fig. 1).

### Metabarcoding

Our metabarcoding data derive from three samplings of the Geumsan reactor, one each in June 2023, January 2024, and April 2024 (“GSJu”, “GSJa”, and “GSAp”, respectively). While VFA concentrations and biogas production were higher in April 2024 than in June 2023 and January 2024, the overall operational conditions were roughly comparable for each sampling (Fig. 1).

**Fig. 2:**
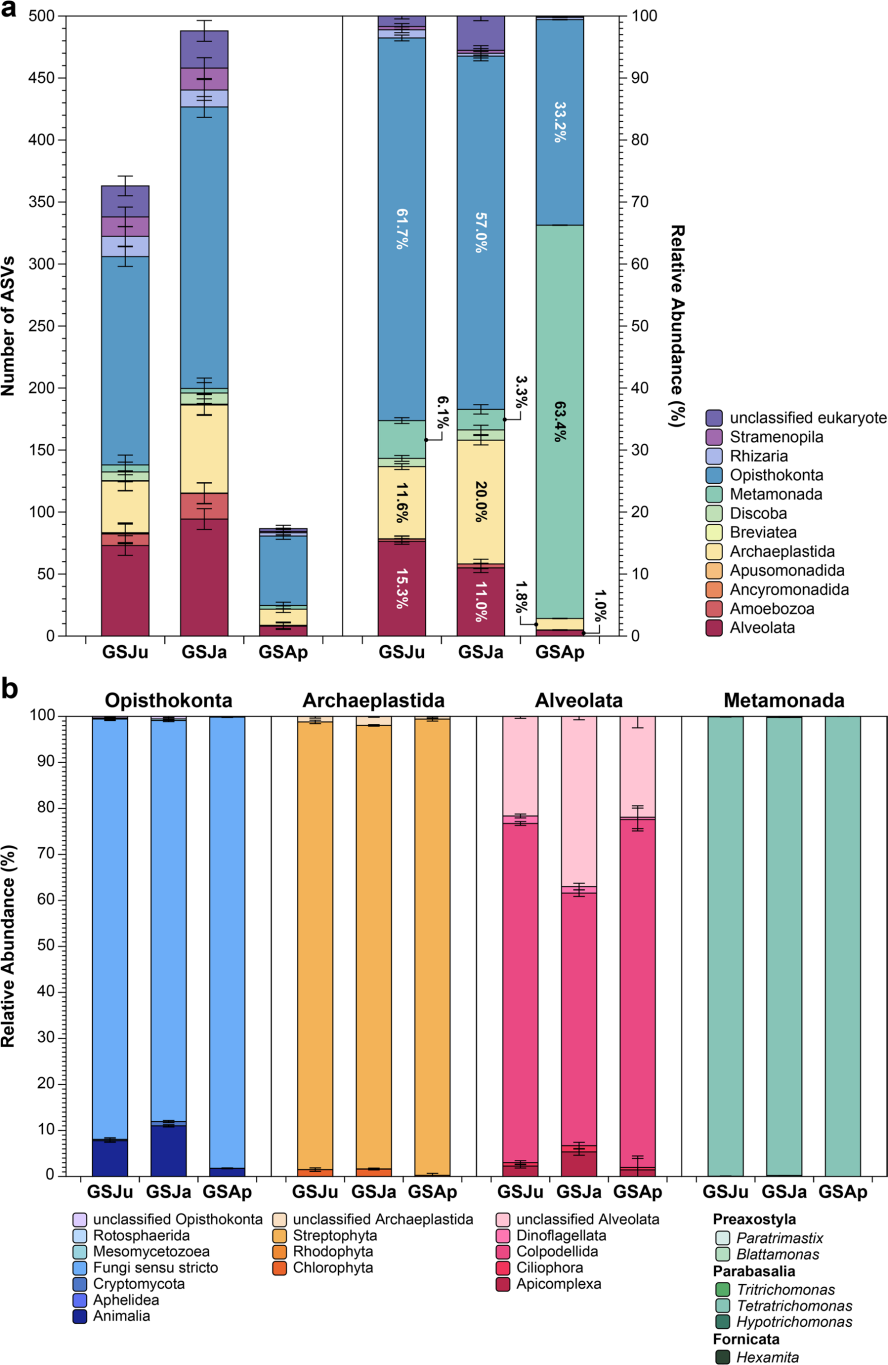
Abundance data from digester at Geumsan over ten months.** a** Kingdom/supergroup-level data. Values for ASVs (left) are absolute numbers; those for relative abundance (right) are proportions of all relevant NGS reads, with percentages shown for most-highly represented groups. **b** Relative abundance data for each of the most-highly represented kingdom/supergroup-level groups: opisthokonts (by kingdom/phylum), archaeplastids (by phylum), alveolates (by phylum), and metamonads (by genus)

The communities from the GSJu and GSJa samples were broadly similar; however, the GSJu sample produced roughly three-fourths as many ASVs as that from GSJa (Fig. 2a, left). The community from the GSAp sample was substantially different, showing less than a fourth of the number of ASVs as the GSJu sample (Fig. 2a, left). The relative abundance of supergroup/kingdom-level taxa shifted between each sample, but again, GSAp had the strongest difference, with metamonads and opisthokonts dominating at the expense of all other taxa (Fig. 2a, right). In the GSJu and GSJa samples, the dominant supergroup/kingdom-level taxa were opisthokonts, archaeplastids, and alveolates (Fig. 2a).

Within opisthokonts and archaeplastids, arthropods (e.g., mites), fungi (e.g., aerobic yeasts), and streptophytes (e.g., cabbage, zucchini, sweet potato), all of which were directly associated with the feed material, such as livestock manure and food waste, dominated in all samples (Fig. 2b). Within alveolates, overall diversity was generally similar across samples, with colpodellids (a parasitic taxon with members derived from the feed material) showing the highest abundance (Fig. 2b). Notably, a substantial proportion of alveolate sequences could not be classified even at the phylum level (Fig. 2b). Within metamonads, *Tetratrichomonas* (including our cultured strains) consistently accounted for the vast majority of individual reads throughout the entire period, exhibiting exceptionally high abundance (Fig. 2b).

**Fig. 3:**
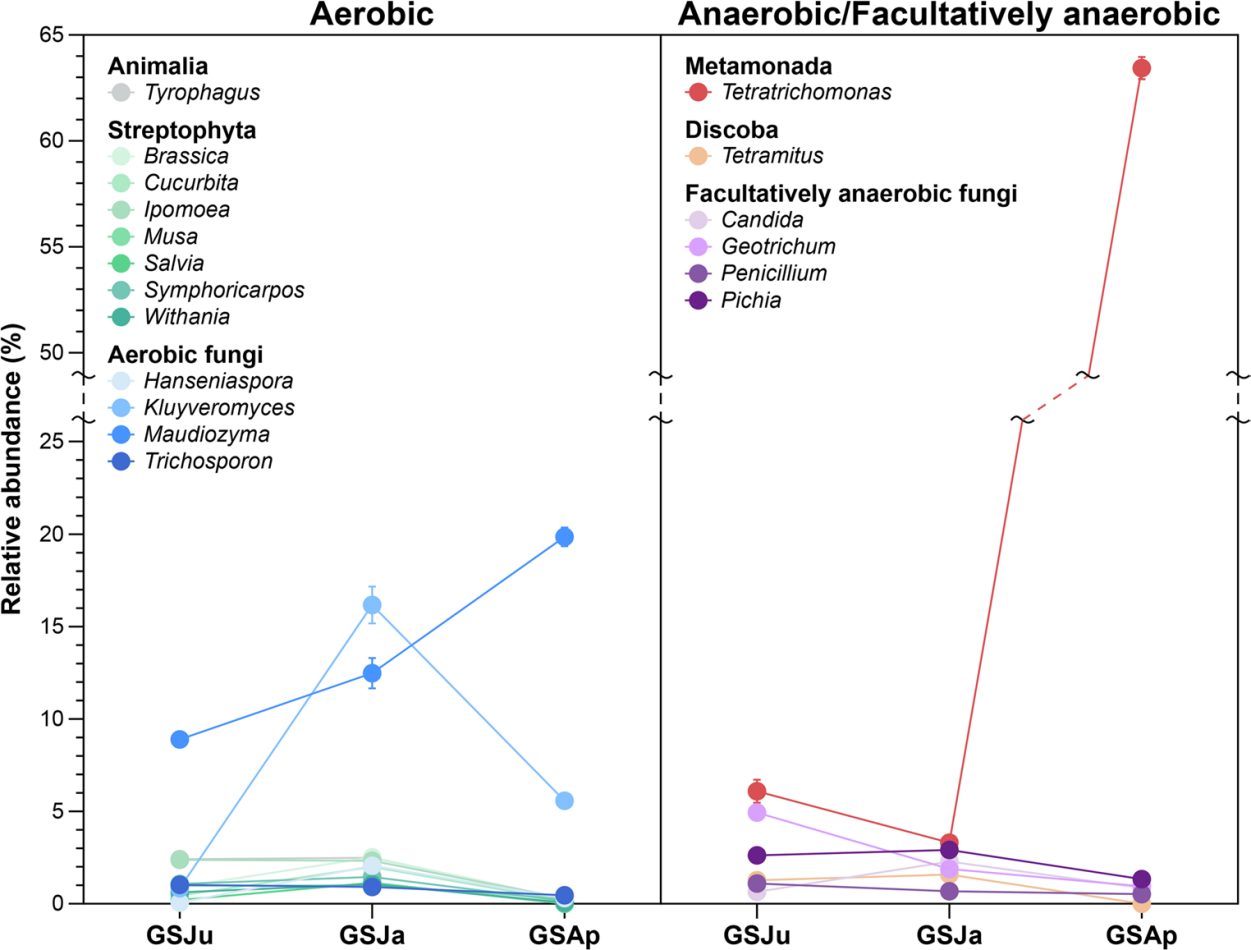
Relative abundance from digester at Geumsan over ten months, broken down by kingdom and genus. The left and right panels indicate the identified aerobic and anaerobic/facultatively anaerobic taxa, respectively. Only taxa both with > 1.0% relative abundance and identifiable to genus are shown (a total of 18 genera). GSJu, GSJa, and GSAp represent sampling times from the Geumsan reactor in June 2023, January 2024, and April 2024, respectively

The 18 genera with relative abundances greater than 1% displayed diverse distribution patterns across the sampling points (Fig. 3). Overall, most major aerobic and anaerobic taxa exhibited noticeable shifts in relative abundance (Fig. 3). Remarkably, the anaerobic *Tetratrichomonas* decreased in relative abundance from GSJu to GSJa, followed by an overwhelming increase in GSAp, accounting for 63.4% of the relative abundance in the latter (Fig. 3, right). The aerobic fungus *Kluyveromyces* peaked in GSJa and was more abundant in GSAp than in GSJu, whereas another aerobic fungus, *Maudiozyma*, showed a gradual increase across the sampling time (Fig. 3, left). The facultatively anaerobic fungi *Geotrichum* and *Penicillium* gradually declined in relative abundance from GSJu to GSAp (Fig. 3, right).

### Characterization of Novel Organisms

Microscopy-related Methods, Results, and Figures of novel organisms are provided in the Supplementary Information; we focus here on molecular phylogeny.

**Fig. 4:**
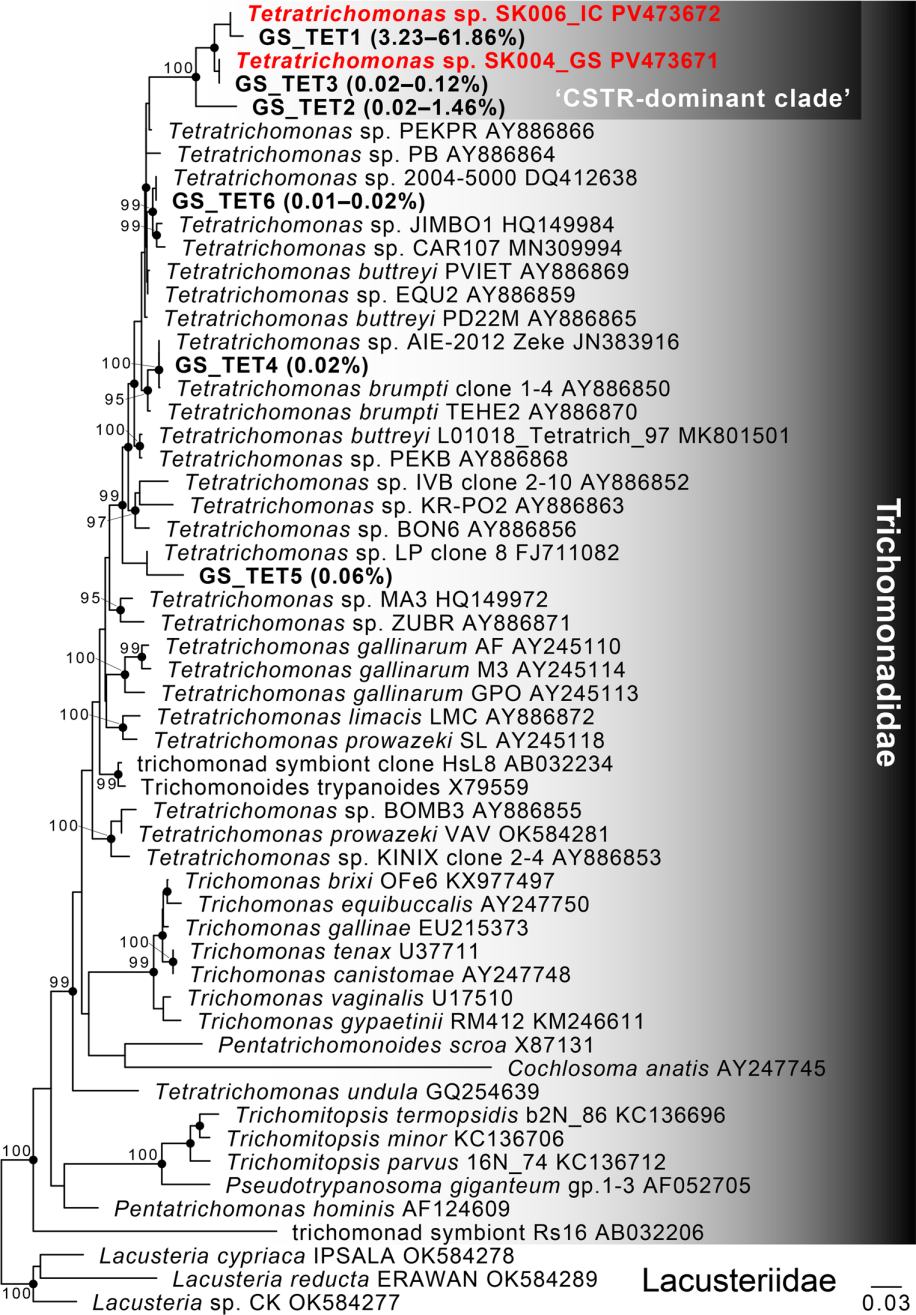
Ribosomal small-subunit RNA phylogeny of Trichomonadidae. Tree was inferred using maximum likelihood and Bayesian inference, rooted with members of Lacusteriidae as an outgroup. Our cultured isolates SK004_GS and SK006_IC are represented in red, while six *Tetratrichomonas*-like ASVs are represented in bold black, with their relative abundance ranges in parentheses. Ultrafast bootstrap support values (≥ 95%) are shown at the nodes. Solid circles denote Bayesian posterior probabilities of 1; posterior probabilities below 0.95 are not shown

The 18S sequences of strains SK002_GS, SK003_GS, and SK004_GS were identical to each other; that of SK004_GS was used to represent all three isolates (details in Supplementary Information). Sequences of isolates SK004_GS and SK006_IC appeared to be similar but not identical to each other. The closest BLASTN matches against GenBank to SK004_GS and SK006_IC sequences returned 95.1% and 94.3% identity, with 100% and 99% coverage, respectively; *Tetratrichomonas buttreyi* (accession AY886865.1) was the closest match to each, while SK004_GS and SK006_IC shared 97.5% identity with one another. The family Trichomonadidae, including our two novel isolates (i.e., SK004_GS and SK006_IC), appears in our analyses as a well-to-maximally supported group (Fig. 4). The only poorly-supported trichomonad genus was *Tetratrichomonas*, which comprised a morass of numerous well-supported species-level clades along with even-more numerous individual sequences, all but a handful of which were from uncharacterized sources. Our two novel trichomonad isolates appeared as a distinct, maximally supported and long-branching clade amongst the *Tetratrichomonas* sequences.

Among the six *Tetratrichomonas*-like ASVs retrieved using the culture-independent approach, three ASVs (GS_TET1, GS_TET2, and GS_TET3) clustered with the SK004_GS and SK006_IC sequences with maximal support values (Fig. 4). Notably, GS_TET1 dominated the eukaryotic community in GSAp, accounting for 61.86% of the total relative abundance. The remaining three ASVs (GS_TET4, GS_TET5, and GS_TET6), all present at low abundances, were affiliated with other *Tetratrichomonas* spp., and were not directly related to our two novel isolates (Fig. 4).

### Correlation Between Operational Factors and Eukaryotic Taxa

**Fig. 5:**
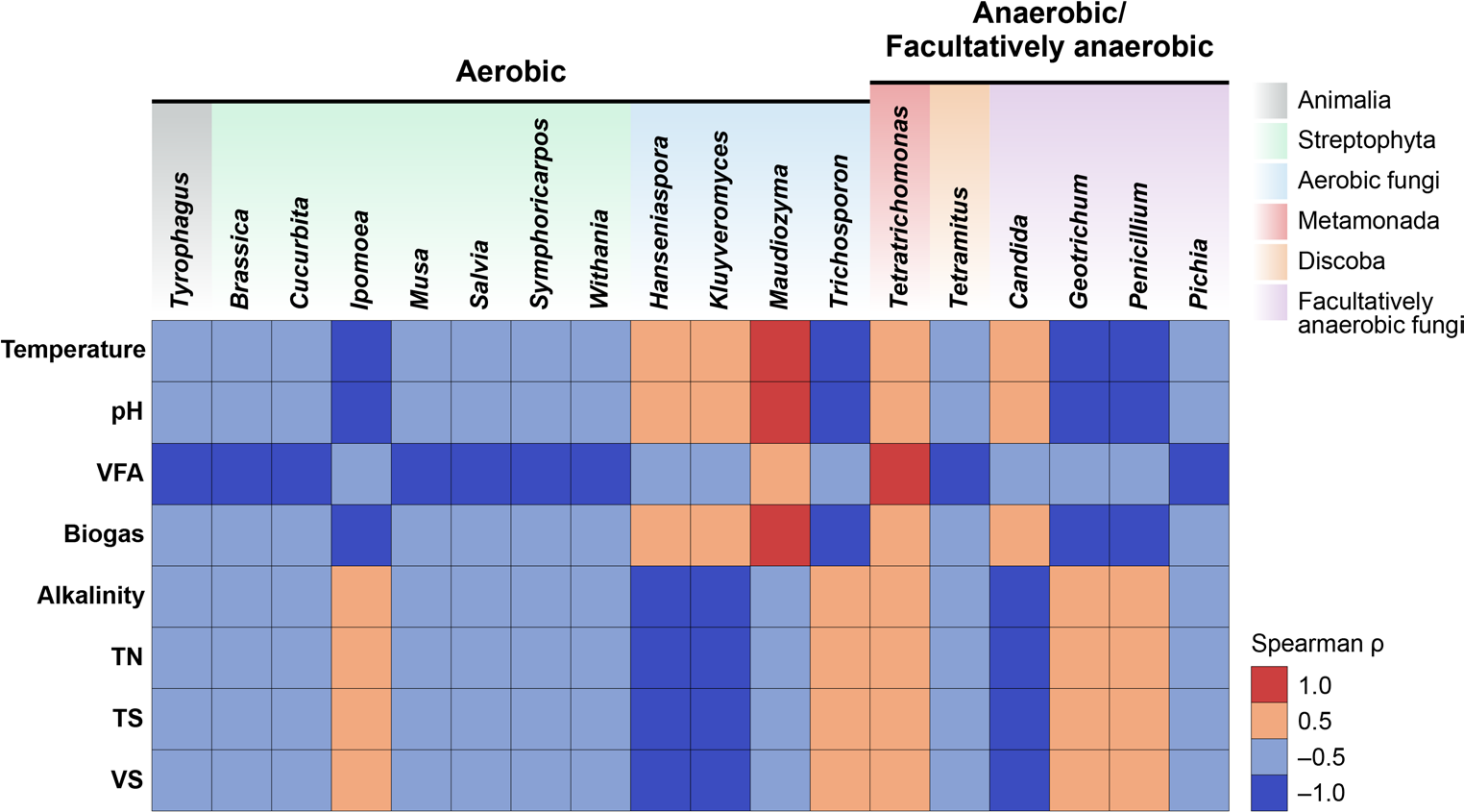
Spearman correlation matrix between operational factors and eukaryotic taxa. The heatmap shows pairwise Spearman correlation coefficients (ρ) between eight operational factors and the 18 eukaryotic genera with > 1.0% relative abundance. Warm colors indicate positive correlations; cool colors indicate negative correlations. Color shading at top indicates the taxonomic kingdom of each genus

Analysis of the correlation matrix suggested potential relationships between operational factors and the relative abundances of eukaryotic taxa, in spite of the limited number of samples (Fig. 5). *Tetratrichomonas* showed consistently positive correlations with all operational factors and exhibited a particularly strong positive correlation with VFA (ρ = 1). In contrast, feed-material-driven-taxa, such as the aerobic mite *Tyrophagus* and most streptophytes (except *Ipomoea*), as well as the facultatively anaerobic discobid *Tetramitus*, showed generally negative correlations with all operational variables, with the strongest negative relationship observed with VFA (ρ = −1). Fungi showed variable relationships with various operational factors. Facultatively anaerobic fungi, including *Candida*, *Geotrichum*, *Penicillium*, and *Pichia*, showed negative correlations with VFA, whereas the aerobic fungus *Maudiozyma* showed positive correlations with VFA and biogas production.

## Discussion

Our study combined isolation and culturing of individual organisms with NGS metabarcoding. Only the former approach allows for detailed characterization of individual strains, while the latter can supply more-objective data on diversity and relative abundance. In this study, we found that most eukaryotic sequences extracted from anaerobic digesters are probably from feed material, as inactive biota (e.g., mites, aerobic fungi, cabbage, zucchini, and sweet potato), while those from eukaryotes culturable under anoxic conditions were generally rare. Intriguingly, the anaerobic *Tetratrichomonas* spp., a metamonad (specifically, phylum Parabasalia), which has previously been found in the intestines of various animals [28, 29], was the only genus prominently detected using both culture-dependent and culture-independent approaches. This genus comprised 3.3–63.4% of the total eukaryotic community in culture-independent analyses (Fig. 3, right). In particular, our results indicate that our two newly obtained isolates represent the dominant *Tetratrichomonas* group in the anaerobic digester system. Our assessment of high relative abundance is probably accurate, given that the 18S rRNA gene of our *Tetratrichomonas* spp. has an average copy number of ~ 80/cell (see Supplementary Information). The rRNA gene copy numbers of ciliates, fungi, and foraminifera have consistently been reported to be higher than those in *Tetratrichomonas* spp. [30–32].

Although the number of samples was limited, the relative abundance of *Tetratrichomonas* showed a positive association with VFA concentrations, which are linked to changes in digester pH and microbial activity, as well as to biogas produced by methanogenic archaea as the end product of anaerobic digestion (Fig. 5). Notably, typical Korean anaerobic digesters, including those at both of our sampling sites, exhibit elevated VFA concentrations in March and April, coinciding with the late-winter to early-spring cleaning of livestock manure storage tanks [33]. Likewise, in this study, the monthly average biogas production during March and April in 2023 and 2024 (2,220–2,583 Nm^3^/day) was higher than that in other months (1,539–2,108 Nm^3^/day; Fig. 1). These observations indicate that *Tetratrichomonas* increased in relative abundance during periods when VFA concentration and biogas production were high. However, the functional role of *Tetratrichomonas* in VFA turnover or biogas production has not been experimentally demonstrated, and therefore these observations do not indicate a direct relationship with digester performance. Moreover, the two predominant fungi that we found in these digesters, *Kluyveromyces* and *Maudiozyma* (= *Kazachstania*), are not known to grow under anaerobic conditions [34, 35], suggesting that they are likely inactive. Other predominant but facultatively anaerobic fungi, such as *Pichia* and *Geotrichum*, may be capable of thriving in Korean anaerobic digesters. However, *Pichia* and *Geotrichum* were consistently less abundant than *Tetratrichomonas* across all metabarcoding samples (Fig. 3).

Comparison of our results to previous findings is hindered by differing methodologies and feed material. Ours is a pilot long-term survey to focus on the mature eukaryotic biota in a CSTR, which we surveyed using the V9 hypervariable region of the 18S gene. Previous metabarcoding work has focused on up-flow anaerobic sludge blanket reactors [4, 36] or other designs [3, 10]; we are aware of only one previous metabarcoding study of a CSTR [2], which focused on its startup phase, and was not focused on eukaryotes (although it did consider them). Protist diversity in anaerobic reactors has been previously probed either using the V4 region of the 18S gene [2, 36], an extended internal region [10], or the entire gene [3]. Only one study of which we are aware assessed diversity using the V9 region; this same work also examined the V4 region, and showed that the two hypervariable regions gave very different results [4]. In addition, ours is also the first investigation of an anaerobic digester to have occurred in Korea, as well as to have used co-digested feed material (in our case, a mixture of ~ 6:1:1 livestock manure, food waste, and sewage sludge, respectively).

The Hirakata et al. [4] study is noteworthy in that it was the first metabarcoding study of a full-scale anaerobic digester to be combined with a culturing study, in this case as a separate follow-up [7]. As with our efforts, Hirakata et al. [7] were able to isolate and culture a parabasalid, albeit from a different genus (*Trichomitus*). They also isolated and cultured a ciliate (*Cyclidium* sp.). Despite successful isolation, their published V4-region data show a fairly constant but also low signal (≤ 1% of all reads) from *Trichomitus* and a very weak signal (~ 0.1%) from *Cyclidium*, suggesting that cultured taxa represented a very minor component of the anaerobic digester biota detectable by metabarcoding. While assessment of overall reactor biota on a population level by microscopic observation may be less practical than metabarcoding analyses, assessment of the contributions of highly represented indicator taxa will still require comparative studies involving cultures derived from anaerobic reactors. We are aware of very few such studies [6, 7, 9, 37], each of which focused on a small number of taxa. Aside from *Tetratrichomonas* spp., our results confirm prior studies’ (e.g., [4, 7]) suggestion that culturable taxa can differ strongly from those identifiable by metabarcoding. This discrepancy is meaningful for better understanding the diversity of active and inactive organisms in anaerobic digesters. Based on the culture-dependent approach, we find that heterotrophic ciliates and parabasalids may be ecologically relevant groups of protists in anaerobic digesters [7].

Ciliates were found to be prominent members of the eukaryotic biota in previous work (e.g., [2–4, 6, 7]), both in microscopic observations and in other metabarcoding studies. In time-series analyses [2, 4], their proportional representation fluctuated, such that individual genera frequently fell below (and often later returned to) detectable levels [4], but even in cases in which the entire ciliate population crashed [2], ciliates nevertheless continued to comprise ~ 0.5% of relative abundance. In contrast, in our study, ciliates accounted for < 0.15% of relative abundance from GSJu (specifically, 0.114%), GSJa (0.149%), and GSAp (0.005%). Correspondingly, under the microscope, we found none of these prominent and readily identifiable organisms in any of our samples from either of the sampled reactors. As stated, though, differences between our and previous studies (including feed material, reactor type, and target sequences for NGS) impede responsible speculation on the reasons for these biotic differences.

There is nothing in the literature that would lead one to anticipate the dramatic turnover in eukaryotes that we observed between our GSJa and GSAp samples. A sudden crash in ciliate population seen by Goux et al. [2] was not accompanied by any likewise sudden increase in the representation of any other known group (rather, the difference was taken up by unidentifiable sequences), and other well-represented lineages remained at comparable proportions before and after the crash. Likewise, a sudden spike in perkinsid sequences and an accompanying increase and subsequent attenuation of the breviate population reported by Hirakata et al. [4] were not also accompanied by any substantial changes in the proportions of other taxa. By contrast, in our data, not only did the proportion of *Tetratrichomonas* spp. increase substantially in absolute terms compared with other samples, but the proportions of all other taxa combined also dropped. This unexpected dynamic is likely associated with interactions between possibly opportunistic heterotrophic eukaryotes and prokaryotes within the anerobic digester environment [7].

*Tetratrichomonas* may affect operational factors indirectly rather than exerting a direct effect. Heterotrophic protists in anaerobic digesters can affect microbial communities through predation, competition, and symbiosis [38]. It is possible that *Tetratrichomonas* preferentially feeds on prokaryotes in anaerobic digesters. This feeding behavior could alter prokaryotic community composition [38, 39]. To assess this (and for other reasons), it would be pertinent to characterize the biological community of the reactor influent in future sampling studies.

We cannot say whether *Tetratrichomonas* maintained its dominance amongst the eukaryotic biota after our final sampling (GSAp), or whether the other biota returned to prior absolute levels or proportions. We note that the operational data show a temporary, roughly-month-long increase in volatile fatty acid and biogas production around the GSAp sampling date, and a decrease in pH afterward (Fig. 1). Despite this fluctuation, the Geumsan reactor continued to operate within normal parameters overall. Therefore, whatever triggered the eukaryotic turnover in Geumsan might not have had a lasting impact on the digester’s overall functionality. As such, it is important to monitor fluctuations of *Tetratrichomonas* spp. in anaerobic digesters more frequently, alongside the prokaryotic community.

## Conclusions

Despite being controlled systems with relatively constrained environmental variability compared to natural ecosystems, we still understand very little about the eukaryotic communities within anaerobic digesters. As detailed above, the eukaryotic complements of these ecosystems vary drastically (compare our results with [2–4, 10, 36]), likely due at least to differences in feed material and seasonal variation. Notably, this study includes the first successful isolation of microbial eukaryotes from Korean mesophilic anaerobic digesters, and the newly obtained isolates were found to be highly dominant within the digester community. Our findings support several of the hypotheses proposed at the outset of this study: the diversity of microbial eukaryotes was different from that reported in previous studies; *Tetratrichomonas* constituted a substantial and recurrent component of the active eukaryotic community in Korean anaerobic digesters; and its relative abundance showed a positive correlation with VFA concentration and biogas production. Further long-term monitoring and experimental studies on *Tetratrichomonas* will be necessary to better understand its ecological role and its potential relevance to biogas production in anaerobic digesters. This pilot study provides an important contribution to our understanding of microbial eukaryotes, such as *Tetratrichomonas*, in anaerobic digesters, and offers substantial potential for future research expansion.

## Supplementary Information

Below is the link to the electronic supplementary material.

Supplementary Material 1 (DOCX 1.19 MB)

## Data Availability

The 18S rRNA gene sequences of SK004_GS and SK006_IC generated and analyzed during the current study are available in the NCBI GenBank repository under the accession codes PV473671 and PV473672, respectively. The amplicon sequencing data are available in the NCBI SRA repository, BioProject PRJNA1247748.
