# Supplementary material for: Characterization of Dominant Heterotrophic Flagellates in Anaerobic Digesters Using Combined Culture-based and Metabarcoding Approaches
Source: Microb Ecol. 2026 Apr 15;89(1):135. doi: 10.1007/s00248-026-02768-5 (PMC13319722; doi:10.1007/s00248-026-02768-5)
Supplement: Supplementary file 1 — Supplementary Material 1 (DOCX 1.19 MB) [file 248_2026_2768_MOESM1_ESM.docx]

**Supplementary information to:**

**Characterization of dominant heterotrophic flagellates in anaerobic digesters using combined culture-based and metabarcoding approaches**

Hyeon Been Lee^a,1^, Aaron A. Heiss^b,1^, Dong Hyuk Jeong^a^, Jinho Cho^c^, ChangWeon Lee^c^, Byung Cheol Cho^d^, Jong Soo Park^a,*^

^a^ Department of Oceanography, Kyungpook National University, Daegu, Republic of Korea

^b^ Microbial Oceanography Laboratory, Kyungpook Institute of Oceanography, Kyungpook National University, Daegu, Republic of Korea

^c^ SK Incheon Petrochem Co., Ltd., Incheon, Republic of Korea

^d^ School of Earth and Environmental Sciences, Seoul National University, Seoul, Republic of Korea

^1^ These authors contributed equally to this work.

^*^ Correspondence: Jong Soo Park
[jongsoopark@knu.ac.kr](mailto:jongsoopark@knu.ac.kr)

**Supplementary methods**

**Microscopy**

Cultures of strains SK004_GS and SK006_IC were imaged using phase contrast and differential interference contrast optics on a Leica DM5500B microscope (Leica, Wetzlar, Germany) with a 100×, NA=1.0 objective. Images were captured using a Leica DFC550 camera and dimensions (N ≥ 20 for all cells and cysts) were measured using FIJI v2.14.0 [1].

For scanning electron microscopy (SEM), a mature culture of strain SK003_GS was concentrated by centrifugation for 10 min at 1,000 × *g* and fixed with 2.5% (v/v) glutaraldehyde (final conc.) in its growth medium in microcentrifuge tubes overnight at 4°C. Fixed cells were washed three times with fresh medium by centrifuging for 10 min at 1,000 × *g*, and postfixed with 2% (w/v) OsO_4_ (final conc.) in medium for 1 hr on ice while settling on glass coverslips coated with 0.01% poly-L-lysine. These were rinsed with fresh medium, 50% medium, and pure water, and then dehydrated through an ethanol series (10%, 30%, 50%, 70%, 80%, 90%, 95% × 2, and 100% × 3, 10 min each step) and dried using a critical point dryer (HCP-2; Hitachi, Tokyo, Japan).

A mature culture of strain SK006_IC was prepared for SEM by concentration for 4 min at 3,000 × *g*, and fixed with 2.5% (v/v) glutaraldehyde (final conc.) in growth medium for 1 hr at RT, then washed with fresh medium three times. Fixed cells were transferred to a 0.01% poly-L-lysine-coated coverslip, and postfixed with 1% (w/v) OsO_4_ (final conc.) for 30 min on ice. After rinsing with fresh medium, 50% medium, and pure water, cells were dehydrated using a graded ethanol series (10%, 30%, 50%, 70%, 90%, 95% × 1, and 100% × 3, 10 min each step) and dried using *tert*-butanol in a freeze dryer (Alpha 1-2 LDplus, Christ, Osterode am Harz, Germany). All specimens prepared for SEM were coated with platinum using an ion-sputter system. Specimens were examined under a SU8220 field emission SEM (Hitachi).

For transmission electron microscopy (TEM), cultures of strains SK004_GS and SK006_IC were concentrated by centrifugation for 10 min at 1,000 × g and fixed with 4% (v/v) glutaraldehyde (final conc.) in their growth medium for 1 h on ice. Fixed cells were washed three times with fresh medium, then post-fixed with 2% (w/v) OsO4 in growth medium for 2 h on ice. Following post-fixation, cells were washed sequentially with fresh medium, 50% medium, and pure water. The fixed cells were entrapped in 2% agarose, cut into ~1 mm blocks, and dehydrated through an ethanol series (10%, 30%, 50%, 70%, 90%, 95%, and 100% × 3, 5 min each step). Agarose blocks were infiltrated with Spurr’s resin (SPI Supplies, West Chester, PA, USA) and polymerized at 70°C for 48 h. Ultrathin sections (~70 nm) were prepared using an ultramicrotome (EM UC7, Leica, Wetzlar, Germany) equipped with a diamond knife (Ultra 35˚, DiATOME, Nidau, Switzerland). Sections were stained with UranyLess EM Stain (Electron Microscopy Sciences, Morgantown, PA, USA), counterstained with lead citrate (Electron Microscopy Sciences), and observed using a HT-7700 TEM (Hitachi) operating at 100 keV.

**Digital PCR**

Digital PCR (dPCR) was used to determine the 18S rRNA gene copy number of strains SK004_GS and SK006_IC. For this, Primer3 [2] was used to design “TET1,” a candidate primer set for the 18S rRNA gene of both strains (Fwd: 5'-CTTCCGCCTGCTAAAATCGC-3'; Rev: 5'-GGTTGACCTGTCTAGCGTTGA-3'). A BLASTN search confirmed that TET1 is a genus-specific primer set for *Tetratrichomonas*. DNA from strains SK004_GS and SK006_IC was extracted using the DNeasy Blood and Tissue Kit (Qiagen, Hilden, Germany) according to the manufacturer’s instructions. The extracted DNA was then used to assess the efficiency of the TET1 primer set with the StepOnePlus Real-Time PCR System (Thermo Fisher Scientific, Waltham, USA). The TET1 primer set successfully amplified both strains with high specificity and efficiency, and without forming primer dimers (not shown).

For cell counting, 1 mL of each culture was fixed with 2% (v/v) borax-buffed formalin (final conc.) and collected on a 25-mm black 0.8-μm-pore polycarbonate filter (Whatman, UK) using a vacuum pump. The fixed cells were stained with 1 mL of DAPI (4',6-diamidino-2-phenylindole dihydrochloride) solution (20 μg/mL) for 10 min and then filtered. The DAPI-stained flagellate cells were counted under UV excitation with a 100× objective lens using a Leica DM5500B microscope equipped with a DFC550 digital camera.

DNA for dPCR analysis was extracted from 1 mL of each culture using the DNeasy Blood and Tissue Kit (Qiagen) according to the manufacturer’s instructions. The 10 μL reaction mixture contained 2 μL of 5× Absolute Q DNA Digital PCR Master Mix (Applied Biosystems, Thermo Fisher Scientific), 1 μL of 10× SYBR Green I Nucleic Acid Gel Stain (Invitrogen, Thermo Fisher Scientific), 0.2 μL of each 10 µM TET1 primer, and 2 μL of extracted DNA. A total of 9 μL of the reaction mixture was loaded into a well of an Absolute Q MAP16 Plate (Applied Biosystems, Thermo Fisher Scientific) and processed in a QuantStudio Absolute Q Digital PCR System (Applied Biosystems). The PCR protocol consisted of an initial preheating step at 96°C for 10 min, followed by 40 cycles of denaturation (96°C for 5 sec) and annealing/extension (60°C for 15 sec). Data analysis was performed using Applied Biosystems QuantStudio Absolute Q Digital PCR Software v6.3.4.

To calculate the 18S rRNA gene copy number per cell, the total copy number per mL obtained from dPCR analysis was divided by the number of cells per mL determined from cell counting. This procedure was repeated, providing an estimated mean value.

**References**

1. Schindelin J, Arganda-Carreras I, Frise E et al (2012) Fiji: an open-source platform for biological-image analysis. Nat Methods 9:676–682. https://doi.org/10.1038/nmeth.2019
2. Untergasser A, Cutcutache I, Koressaar T et al (2012) Primer3—new capabilities and interfaces. Nucleic Acids Res 40:e115. https://doi.org/10.1093/nar/gks596

**Supplementary results**

**Microscopy of Novel Organisms**

*Light microscopy of SK004_GS and SK006_IC.* Unfortunately, SK002_GS and SK003_GS were lost in the course of a power outage; however, their appearance and 18S rRNA gene sequences were identical to those of the successfully maintained isolate SK004_GS. Cells of strains SK004_GS and SK006_IC appeared fundamentally similar to one another (Fig. S2a–f). The principal difference between them was in size, their main cell bodies being 8.0 ± 1.4 (mean ± std; Fig. S2a–d) and 9.2 ± 2.5 (Fig. S2e, f) µm long, respectively, although the ranges overlapped substantially. Under optimal conditions, four anterior flagella could be made out (arrowheads in Fig. S2f). Cells were plastic but generally pyriform, about 1.5× as long as wide. Both strains had cell-length undulating membranes (UMs: arcs in Fig. S2a, b, d–f), and under exceptional conditions, the posterior flagellum could be seen to extend beyond them (not shown). Posteriorly protruding axostyles were frequently obvious (asterisks in Fig. S2a, c–f).

*Scanning electron microscopy of SK003_GS* (= *SK002_GS and SK004_GS*; see above) *and SK006_IC.* As with light-microscopic observations of these cells, the two strains generally appeared very similar to one another under the SEM (Fig. S2g–m). Measurements of cells were about half of those taken under light microscopy, although most seemed to be in lifelike condition otherwise. Protruding axostyles appeared either prominently (e.g., Fig. S2h) or not at all (e.g., Fig. S2g); when they were visible, they featured a pronounced proximal tapering and a long, slowly tapering distal region, on average a little wider than a flagellum, usually with a pointed end (asterisks in Fig. S2h–l). Undulating membranes were readily identifiable (arrows in Fig. S2h–l), although most appeared to terminate without an extension of the posterior flagellum (e.g., Fig. S2m). Most cells of SK003_GS had four anterior flagella (Fig. S2g, h), but only three flagella could be confirmed in some otherwise well-preserved cells of that strain, and cells of SK006_IC were seldom seen with more than three.

*Transmission electron microscopy of SK004_GS and SK006_IC*. TEM showed that both strains possessed hydrogenosomes characterized by a homogeneous matrix and the absence of cristae (Fig. S3). However, no methanogenic endosymbionts were observed in either strain (Fig. S3).

**Estimation of 18S rRNA Gene Copy Number in *Tetratrichomonas***

To determine whether the genus *Tetratrichomonas*, which showed high read counts in the Geumsan reactor, is truly abundant, or if this observation is biased by a high 18S rRNA gene copy number per organism, digital PCR was performed on strains SK004_GS and SK006_IC using a *Tetratrichomonas*-specific primer set (TET1). Despite belonging to the same genus, strains SK004_GS and SK006_IC had slight differences, with approximately 70 and 90 copies per cell, respectively. Therefore, the 18S rRNA gene in the genus *Tetratrichomonas* overall was estimated to have approximately 80 copies.

**Supplementary figure**

**Fig. S1 Rarefaction curves of amplicon sequence variants (ASVs) from triplicate samples collected in June 2023 (GSJu), January 2024 (GSJa), and April 2024 (GSAp).**

**
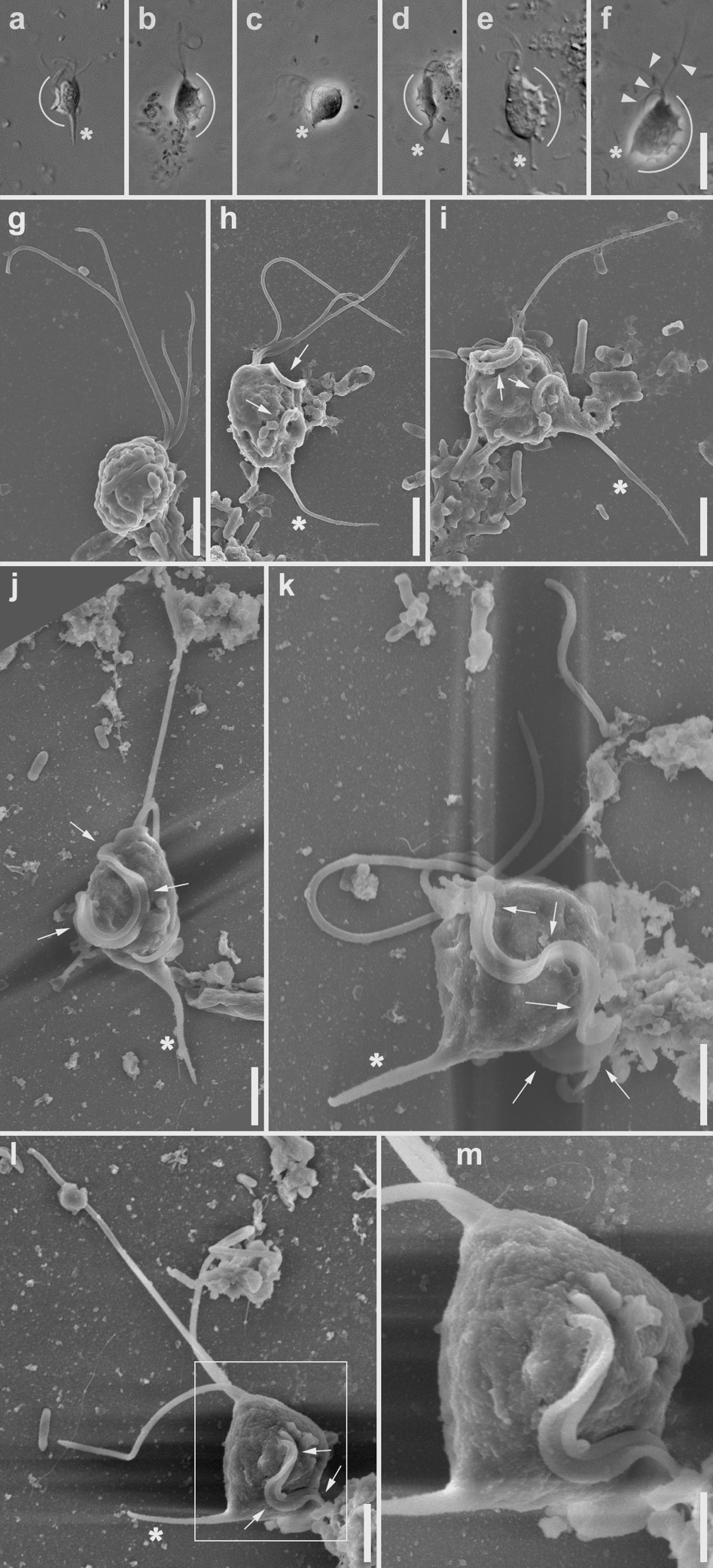
**

**Fig. S2 Microscopy of strains SK003_GS, SK004_GS, and SK006_IC.** **a**–**d** Differential interference contrast (DIC, panel **a**) and phase contrast (PC, **b**–**d**) of cells of strain SK004_GS. Morphology of cells in **a**, **b** is typical, in **c** is almost spherical, and in **d** is long and flexible. Protruding posterior axostyle is long and gradually tapering in **a**, short and strongly tapering in **c**, and flexible in **d**. Undulating membrane (UM) is prominent to left of cell in **a**, **d**, and to right of cell in **b**. **e**–**f** DIC **e** and PC **f** images of same cell of strain SK006_IC. Cell has typical morphology. Note four anterior flagella visible in **f**. **g**–**i** Scanning electron microscopy (SEM) of cells of strain SK003_GS. Note four anterior flagella visible in **g**, posterior protruding and somewhat flexible axostyle in **h**, **i**. UM is prominent to right of cell in **h** and on top of cell (towards viewer) in **i**. **j**–**m** SEM of cells of strain SK006_IC. **m** is magnified image from area indicated by box in **l**. Note prominent axostyle and UM in all images; note especially connection between UM and cell surface in **m**. Annotations: arcs — undulating membrane; arrows — prominent parts of undulating membrane; arrowheads — flagella; asterisks — axostyle. Scale bars: 10 µm in **f** for **a**–**f**, 2 µm in **g**–**l**, 1 µm in **m**.


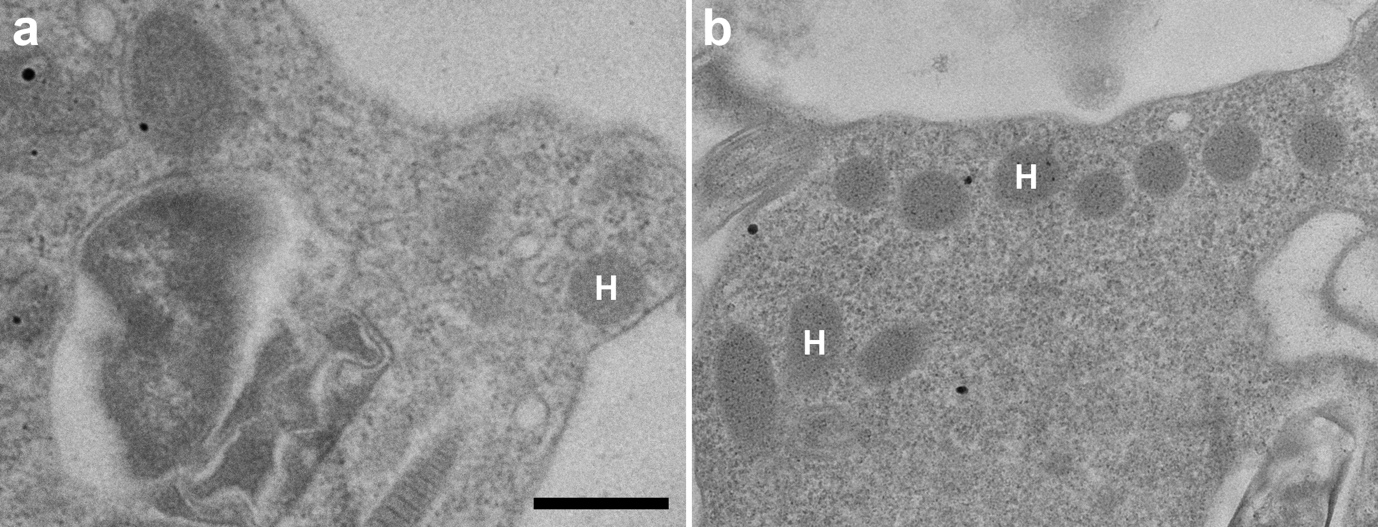


**Fig. S3 Transmission electron microscopy (TEM) of SK004_GS and SK006_IC.** TEM image of strain SK004_GS (**a**) and SK006_IC (**b**). Hydrogenosomes (H) are present in both strains. No methanogenic endosymbionts were observed. Scale bar: 500 nm.
